# Clinical trials of investigational agents for IPF: a review of a Cochrane report

**DOI:** 10.1186/1465-9921-14-S1-S4

**Published:** 2013-04-16

**Authors:** Luca Richeldi

**Affiliations:** 1 Luca. Richeldi, Centre for Rare Lung Disease, University of Modena and Reggio Emilia, Modena, Italy

**Keywords:** Idiopatic Pulmonary Fibrosis (IPF), investigational agents, meta-analysis

## Abstract

The magnitude of treatment effect can be assessed by a number of methods. One reliable method of collectively analysing data from randomised clinical trials is that used in Cochrane reviews. These systematic reviews identify and analyse the available evidence using the reliable method of meta-analysis. These often combine data from studies to provide robust evaluations of overall treatment effects. In 2003, a review of data from studies of corticosteroid use in IPF patients found no evidence of a treatment effect. Similarly, very little evidence was found to support the use of immunomodulatory agents. A recent update of these Cochrane reviews failed to identify any new evidence supporting the use of corticosteroids in IPF. However, a review of non-steroid agents for the treatment of IPF identified data from 15 RCTs that was suitable for analysis. Two trials of interferon gamma-1b were pooled and analysed, but no treatment effect was observed in terms of survival. Meta-analysis of three Phase III studies of pirfenidone treatment in IPF patients suggested that progression-free survival was significantly increased by 30%, demonstrating a reduction in the decline of lung function in IPF patients. In addition, there are numerous ongoing trials investigating potential therapeutic agents which provides hope for IPF patients and their doctors.

## Introduction

It is clear that treatment decisions and the clinical management of patients with idiopathic pulmonary fibrosis (IPF) should be based primarily on the findings of randomised controlled trials, and also, to a certain extent, expert opinion. Since 2004 there has been an increase in the number of clinical trials investigating the treatment of IPF, with 13 randomised clinical trials (RCT) designed and completed during the last eight years. These trials included the use of nine different potential drugs, and are encouraging in terms of the research effort focusing on trying to find an effective treatment for this disease. As the number of clinical trials completed increases, the question then is how best to analyse the data from these studies to determine a treatment effect.

## The Cochrane meta-analysis

There are numerous ways to analyse data, and one of these is the systematic evaluation of available data. This process was pioneered by Cochrane in the 1970s, who stressed the importance of using data from randomised, controlled trials as these are likely to provide more reliable evidence than other sources. The “Cochrane Collaboration” was subsequently established, with the aim of analysing existing data on various treatment effects. The systematic reviews are published in the *Cochrane Library* and identify and appraise the available evidence in a treatment area, using the powerful method of meta-analysis [1]. Cochrane reviews are based on a systematic analysis of all published data, for a given treatment. The identification of data for inclusion in a specific meta-analysis is based on predefined criteria. The statistical approach of meta-analysis allows combining of data from relevant clinical trials and thus provides precise estimates of the effects of a given treatment in a specific patient population. Meta-analyses are typically conducted on a single study endpoint and graphically depicted as “forest plots” [2], which show the effect of different studies on the given endpoint.

A key example of the importance of meta-analyses is that published by Antman et al. (1992) [3] on the effects of oral β-blockers for the secondary prevention of mortality in patients surviving a myocardial infarction. This study showed that a number of trials on this specific topic were performed without need, as a prior meta-analysis would have determined the benefit of β-blockers in treating heart failure patients, therefore avoiding the need for numerous placebo-controlled trials that were subsequently performed, exposing patients to treatment with placebo rather than an active treatment.

## Cochrane meta-analyses in IPF treatment

In 2000 the American Thoracic Society (ATS) and European Respiratory Society (ERS) published an international consensus statement with guidelines for the diagnosis and treatment of IPF [4]. This recommended that patients with IPF should be treated with a combined therapy of corticosteroids and an immunosuppressive agent (e.g. azathioprine or cyclophosphamide) until adequate studies had been performed to define the best treatment for IPF. Three years later, we conducted a Cochrane review of the use of corticosteroids in IPF [5]. This analysis showed that there were no existing placebo-controlled clinical trials that had assessed the efficacy of corticosteroid therapy in IPF patients. Therefore, no existing evidence supported the efficacy of corticosteroids for in the treatment of IPF patients.

We then did the same review on the use of immunomodulatory agents in IPF in 2003 [6]. Four randomised, controlled clinical studies were suitable for a meta-analysis [7-10]; however, these studies used four different immunosuppressive agents (cyclophosphamide, azathioprine, colchicine and interferon gamma-1b) and so a meta-analysis was not possible. The authors concluded that there was little evidence to justify the routine use of any immunosuppressive agent (or in fact any non-corticosteroid agent) in the management of IPF at that time.

In 2010, the Cochrane meta-analysis on the use of corticosteroids for IPF was updated [11]. However, seven years after the publication of the first Cochrane review on the use of corticosteroids in IPF, there was still no evidence to support the efficacy of corticosteroids in the management of IPF, but there was also no evidence to rule out the use of corticosteroids in IPF [11]. Therefore clinicians were left with continued uncertainty over the use of these agents in IPF. Since 2003, no placebo-controlled trials of corticosteroids in IPF had been undertaken, probably due to the fact that there was simply no previous clear evidence to show that they were, or were not, an effective treatment option. However, the recent PANTHER-IPF Study has suggested that there was an increased risk of death and hospitalizations in patients with IPF who were treated with a combination of prednisone, azathioprine, and NAC, as compared with placebo [12].

In 2010, a systematic search was conducted to identify RCT that investigated the use of non-steroid agents for the treatment of IPF. This then became the subject of a further Cochrane Review [13]. This systematic search found that, in 2010, thirteen RCT could be included. Contact with pharmaceutical companies and fellow researchers led to the identification of two other suitable clinical trials which would have been published soon after the timing of the analysis, allowing their inclusion. The analysis of endpoints and quality of the methodology of these fifteen trials reduced the number to seven that were eligible for meta-analyses: only the antifibrotic agents interferon gamma-1b and pirfenidone were evaluated in more than one trial and, therefore, were potentially eligible for two separate meta-analyses.

Combining the data from the two RCT on interferon gamma-1b [14,15] in a meta-analysis, using the clinical endpoint of overall survival, showed that there were no statistically significant differences in mortality between interferon gamma-1b and placebo. Interestingly, the larger of the two trials, published by King et al., was negative for efficacy in terms of the overall survival endpoint [14], whereas the smaller trial almost demonstrated statistical significance [15].

**Figure 1:**
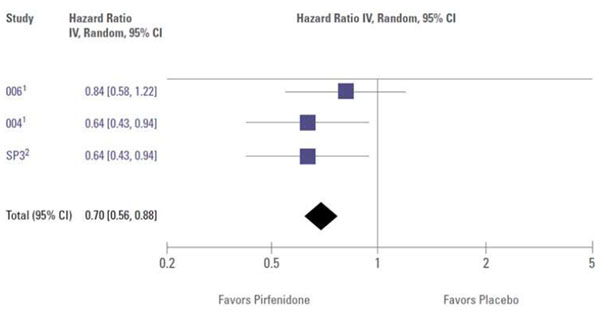
**Meta-analysis of progression-free survival with pirfenidone in IPF** (Adapted from: 1. Spagnolo P, Del Giovane C, Luppi F, *et al. Cochrane Database Syst Rev* 2010, **9:**CD003134. 2. Noble PW, Albera C, Bradford WZ, *et al. Lancet* 2011, **377:**1760-1769. 3. Taniguchi H, Ebina M, Kondoh Y, *et al*. *Eur Respir J* 2010, **35:**821-829.)

For the treatment effect of pirfenidone, the three clinical trials that were eligible for analysis, i.e. the two large, international, randomized CAPACITY (004 and 006) trials [16] and the Japanese SP3 trial,[17] did not have a common primary endpoint (FVC and VC, respectively). However, the identification of data for inclusion in a specific Cochrane meta-analysis is based on pre-defined criteria. Progression-free survival (PFS) data, defined as either death or 10% decrease in FVC, was used as a secondary endpoint in the CAPACITY studies and the Japanese study published by Taniguchi et al.[17] Definitive evidence of clinical efficacy in a Phase 3 trial is best shown by a beneficial impact on a clinically meaningful endpoint—that is, an endpoint that directly measures how a patient feels (symptoms), functions (the ability to perform activities in daily life), or survives. For patients with IPF, there are currently no validated surrogate endpoints [18]. Nevertheless, PFS data from these studies were considered suitable for a meta-analysis based on these criteria. The overall result of this meta-analysis showed that treatment with pirfenidone reduced the risk of disease progression by 30% (HR 0.70, 95% CI 0.56 to 0.88; Figure 1) [13]. This highlights that pirfenidone is the only drug to date that has shown a significant effect on progression free survival, defined as either death or 10% FVC decrease, compared with placebo in patients with IPF.

The number of clinical trials investigating treatments for IPF is expected to continue to increase, and the use of multiple different drugs in the treatment of IPF might be observed in the future. There are multiple on-going trials investigating potential therapeutic agents acting on various different targets. This is very encouraging for IPF patients and doctors, in terms of the research effort focused on finding a suitable treatment.

## Competing interests

Dr. Richeldi reports receiving consulting fees from Boehringer Ingelheim, Intermune, Celgene and Gilead, along with lecture fees from Intermune.

## Acknowledgements

Publication of this supplement was supported by IntraMed Communications with funding from InterMune, AG. InterMune is the manufacturer of pirfenidone, a product mentioned in this article. The supplement originates from presentations given at the “AIR Event: Advancing IPF Research. Working together to translate IPF research into practice” held in Berlin in November 2011. The publication was proposed by IntraMed Communications and developed in consultation with the journal. All articles in the supplement have undergone the journal’s standard peer review process.

## Declarations

This article has been published as part of *Respiratory Research* Volume 14 Supplement 1, 2013:IPF in 2011 – Key updates on guidelines and therapeutics. The full contents of the supplement are available online at http://respiratory-research.com/supplements/14/S1. Publication of this supplement was supported by IntraMed Communications with funding from InterMune, AG. InterMune is the manufacturer of pirfenidone, a product mentioned in this article. The supplement originates from presentations given at the “AIR Event: Advancing IPF Research. Working together to translate IPF research into practice” held in Berlin in November 2011. The publication was proposed by IntraMed Communications and developed in consultation with the journal. All articles in the supplement have undergone the journal’s standard peer review process.
